# Clinical characteristics of lung abscess by red complex bacteria infection: a case report and literature review

**DOI:** 10.3389/fmed.2026.1861751

**Published:** 2026-07-03

**Authors:** Zhiyang Xu, Lian Xu, Jinliang Liu, Lantian Pang, Lexin Xia

**Affiliations:** 1 Department of Infectious Diseases, The Second Affiliated Hospital, Zhejiang University School of Medicine, Hangzhou, China; 2 Department of Infectious Diseases, Zhejiang Cancer Hospital, Hangzhou Institute of Medicine (HIM), Chinese Academy of Sciences, Hangzhou, China

**Keywords:** lung abscess, metagenomic next-generation sequencing (mNGS), Porphyromonas gingivalis, Tannerella forsythia, Treponema denticola

## Abstract

**Background:**

*Treponema denticola, Porphyromonas gingivalis* and *Tannerella forsythia* are common oral pathogens collectively referred to as the “red complex bacteria”, serve as crucial periodontopathic agents. Owing to the challenges associated with anaerobic culture, their contribution to lower respiratory tract infections, especially lung abscess, is often undervalued. Metagenomic next-generation sequencing (mNGS) has evolved as a potent instrument for the identification of fastidious organisms.

**Case presentation:**

A 63-year-old male with chronic cough and hemoptysis was admitted to our hospital. Chest computed tomography showed an indeterminate space-occupying lesion in the right upper lobe, and repeated sputum cultures were negative. Lung cancer was the primary consideration, so a CT-guided percutaneous core needle biopsy of the lung lesion was performed. Nevertheless, the pathology favored inflammation over lung cancer, leading us to continue investigating the causative pathogen. Following mNGS analysis of the puncture biopsy tissue, *Treponema denticola* and *Porphyromonas gingivalis* were detected. Both organisms belong to the red complex bacteria, closely associated with periodontitis that the patient had. Intravenous piperacillin-tazobactam followed by oral amoxicillin-clavulanate was prescribed. The patient recovered and subsequent chest computed tomography confirmed the improvement.

**Conclusions:**

This case highlights the role of oral red complex bacteria in culture-negative chronic lung abscesses. mNGS is a crucial diagnostic tool for identifying these fastidious anaerobes, enabling targeted therapy and improving clinical outcomes.

## Introduction

Lung abscess, characterized by the formation of one or more cavities in the lung tissue, is a severe, necrotizing pulmonary infection frequently caused by the aspiration of polymicrobial oral flora (1). Among these oral microorganisms, the “red complex” bacterial species, which encompass *Treponema denticola, Porphyromonas gingivalis* and *Tannerella forsythia*, are well-recognized anaerobic pathogens and principal pathogens of periodontitis (2). This bacterial group colonizes dental plaque biofilms and can translocate to the lower respiratory tract through aspiration (3). As anaerobic bacteria are common pathogens in lung abscess but difficult to culture via conventional methods, their involvement is often underrecognized, resulting in false-negative outcomes and delayed or inappropriate antimicrobial treatment. Therefore, the accurate identification of causative anaerobic pathogens remains a substantial clinical challenge. The advent of metagenomic next-generation sequencing (mNGS) has revolutionized pathogen detection in infectious diseases, particularly useful for anaerobic and polymicrobial infections (4, 5).

Although isolated cases of lung abscesses caused by *P. gingivalis* have been sporadically reported (6), pulmonary infections explicitly attributed to *T. denticola*, either alone or in synergy with *P. gingivalis*, are exceptionally rare in the literature. Herein, we report a detailed case of a chronic lung abscess caused by co-infection with *T. denticola* and *P. gingivalis* diagnosed via mNGS, and review related literature to elucidate the clinical characteristics, diagnostic strategies, and therapeutic considerations for such infections, providing new insights for the clinical diagnosis and treatment of respiratory infectious diseases complicated by periodontitis.

## Case presentation

A 63-year-old male was admitted to our hospital on November 24, 2025, with a 2-month history of cough and a 1-month history of hemoptysis. He reported no fever, chills, night sweats, or significant weight loss. He was a former smoker with a 35-pack-year history and had quit 5 years earlier. He denied recent dental procedures, alcohol abuse, or history of aspiration. The patient received intravenous piperacillin-tazobactam and voriconazole for approximately 20 days at the local hospital, but clinical improvement was limited. Subsequent chest CT examination suggested the potential presence of lung cancer. Consequently, the patient was referred to our department for treatment.

On admission, vital signs were normal. Laboratory tests showed a normal white blood cell (WBC) count of 6.9 × 10^9^/L with 63.4% neutrophils, a normal C-reactive protein (CRP) of 3.95 mg/L, and within increased erythrocyte sedimentation rate (ESR) (103 mm/h) and normal range levels of procalcitonin (0.09 ng/ml), and IL-6 (7.99 pg/ml). Serum tumor markers, including CEA, CYFRA21-1, NSE, and SCC, were within the normal range. Omadacycline was empirically administered.

**Figure 1:**
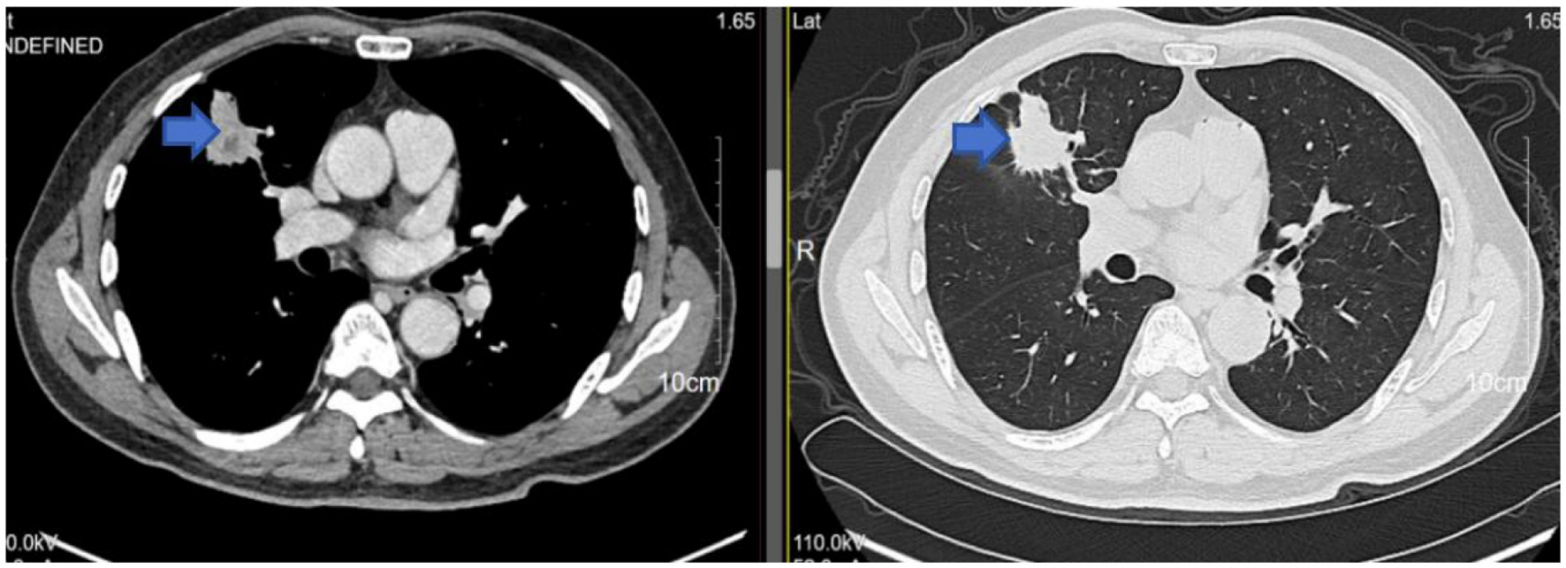
Chest CT scans on admission. The chest CT scan on admission (November 25) revealed spiculated mass in the right upper lobe with surrounding consolidation and minimal liquefactive necrosis.

Contrast-enhanced chest computed tomography (CT) on November 25 showed a 30.8 × 37.4 mm spiculated mass in the right upper lobe, with surrounding consolidation and a small area of liquefactive necrosis (Figure 1). No pleural effusion was present. The images suggested possible lung cancer, and repeated sputum cultures showed no bacterial growth. Thus, lung cancer was initially considered. Therefore, a CT-guided percutaneous core needle biopsy of the lung lesion was performed on November 26, 2 days after his admission. However, histopathological examination of the biopsy tissue showed acute and chronic suppurative inflammation with necrosis, suggestive of a lung abscess, thereby making malignancy less likely. As previous sputum cultures were all negative, the punctured tissues were submitted for mNGS.

**Figure 2:**
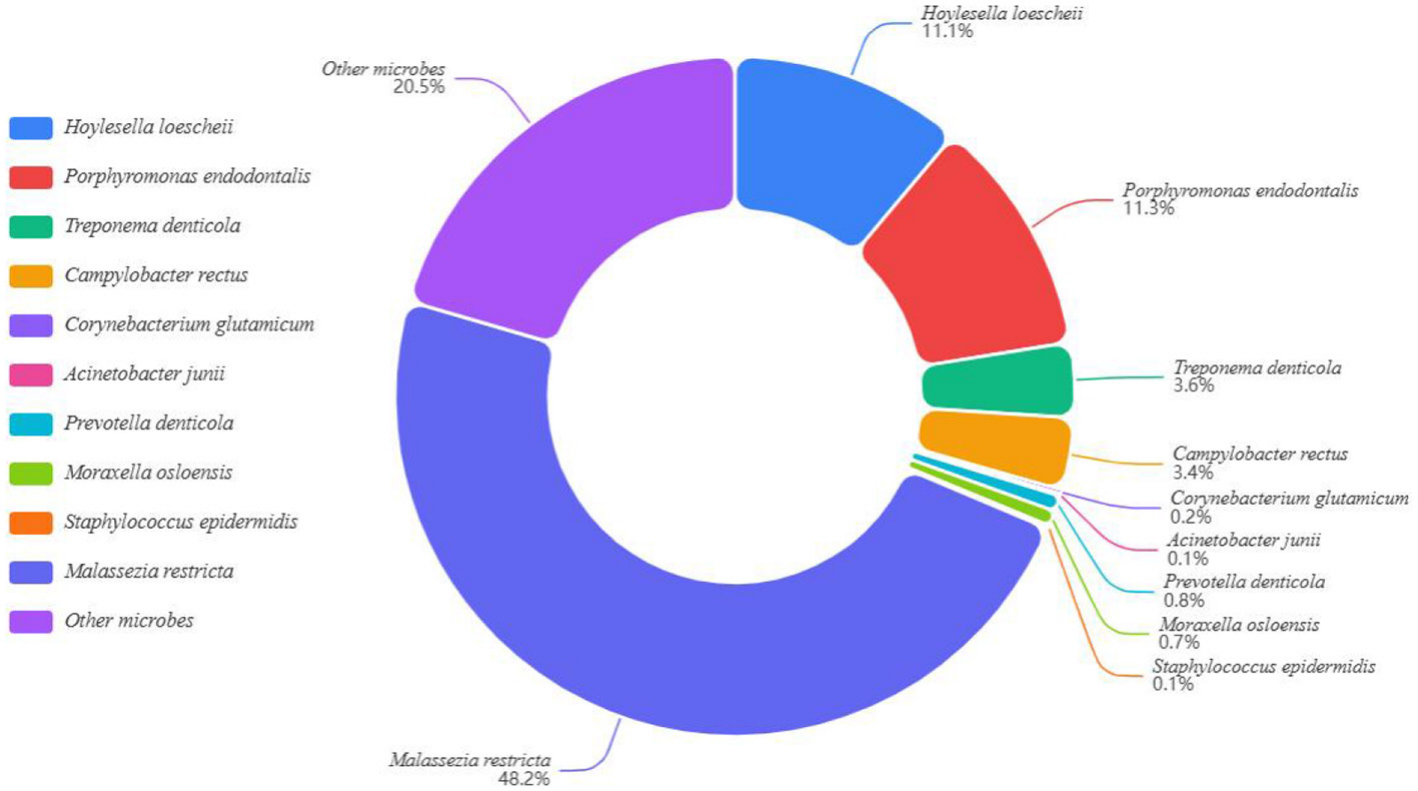
Relative abundance of major organisms detected by biopsy mNGS.

The mNGS analysis revealed *Treponema denticola* (1,730 reads, relative abundance 11.1%) and *Porphyromonas gingivalis* (1,374 reads, 11.35%). No other pathogenic bacteria, fungi, or viruses were detected (Figure 2). Periodontal examination confirmed generalized severe periodontitis with bleeding on probing and subgingival calculus.

**Figure 3:**
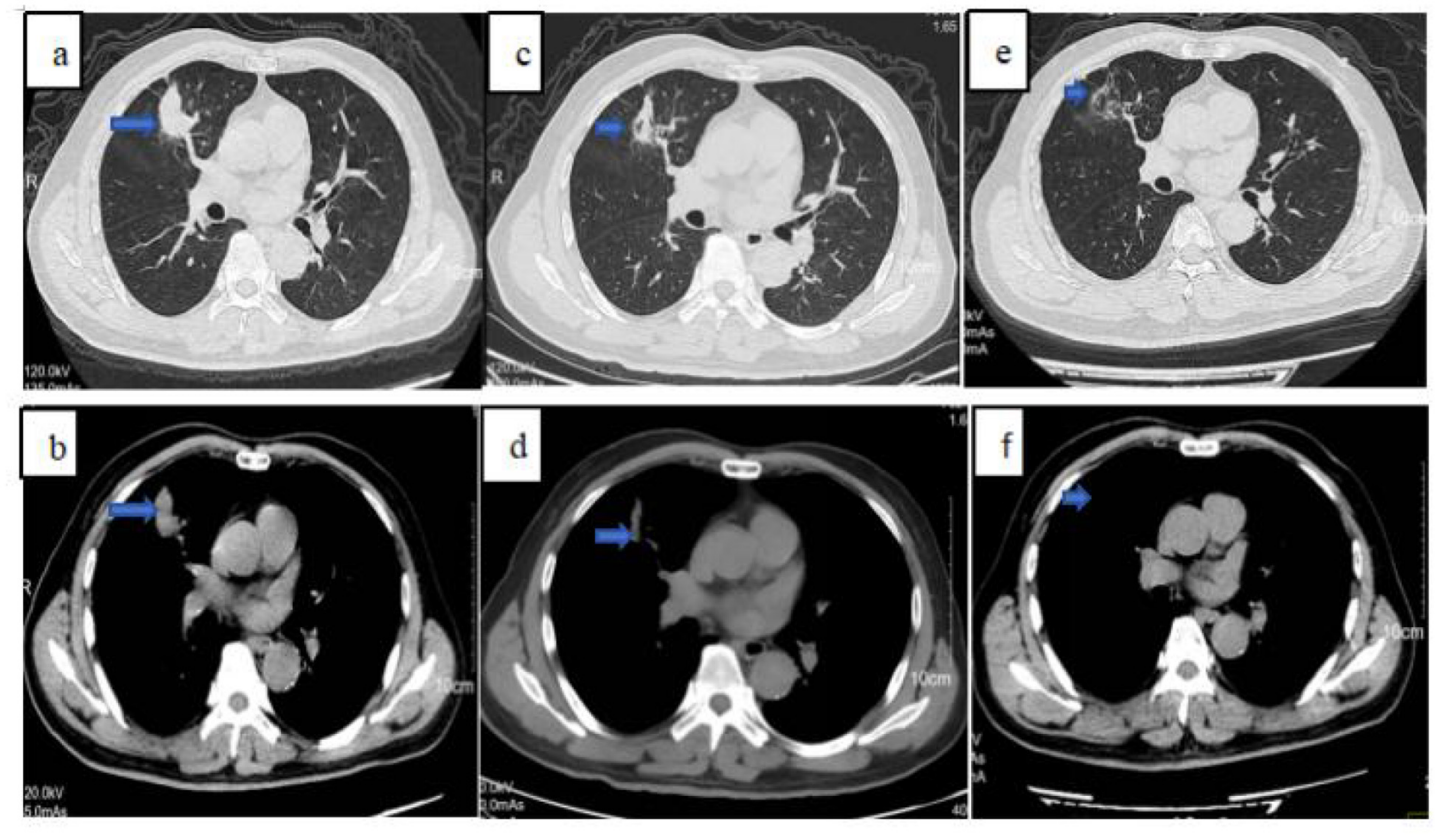
Outpatient follow-up chest CT results. Outpatient chest CT re-examination on December 27, 2025 **(a, b)**, January 24, 2026 **(c, d)** and February 27, 2026 **(e, f)**.

**Figure 4:**
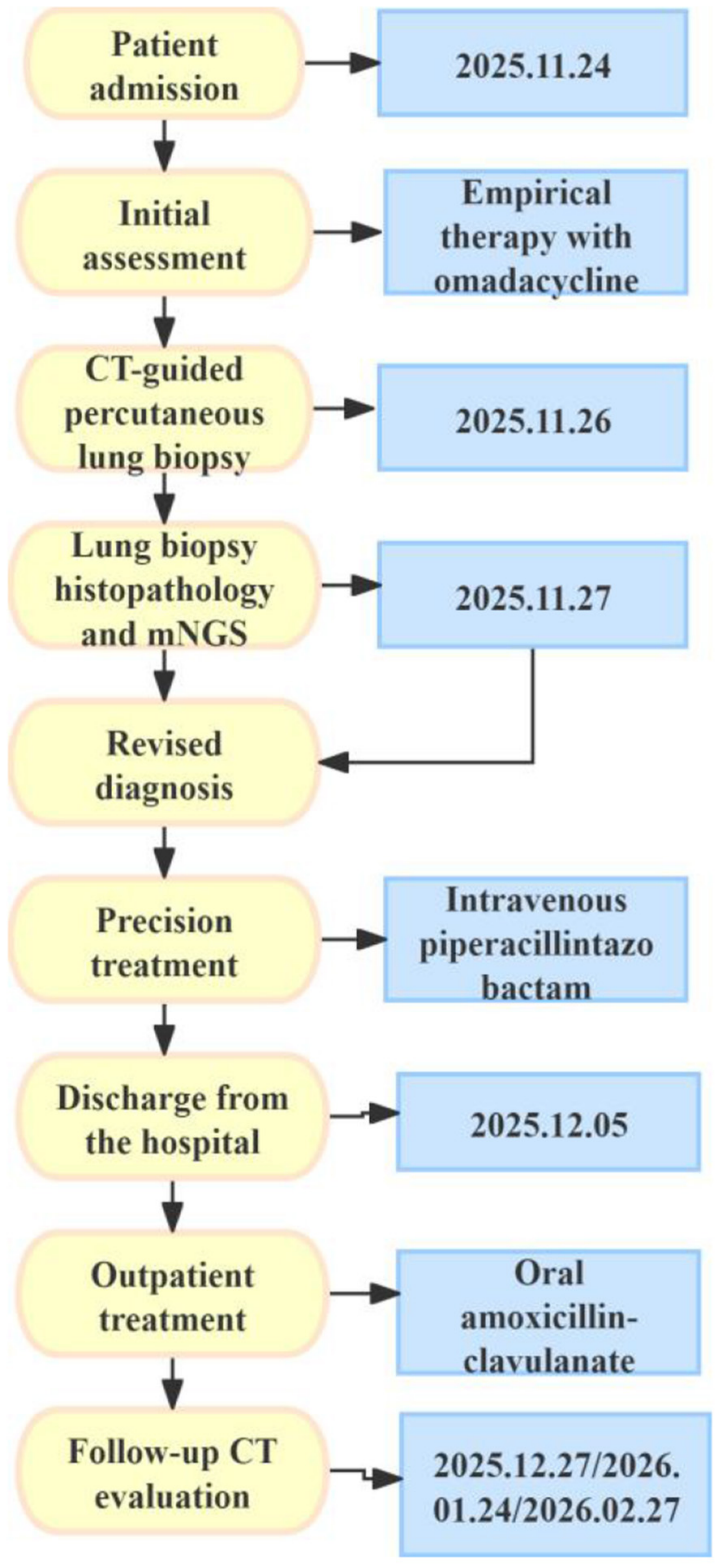
Flowchart depicting the episode of care.

Antibiotics were adjusted to intravenous piperacillin-tazobactam 4.5 g every 8 h for 2 weeks, followed by oral amoxicillin-clavulanate 875 mg/125 mg twice daily for 12 weeks. The patient was also instructed on improved oral hygiene with chlorhexidine mouthwash. His cough and hemoptysis resolved within 1 week. Repeat CT at 3 months showed near-complete resolution of the mass, leaving a small residual fibrotic scar (Figure 3). The process of diagnosis and treatment is presented in Figure 4.

## Discussion

In this case, we report a culture-negative chronic lung abscess caused by red complex anaerobes *T. denticola* and *P. gingivalis* diagnosed by mNGS. This case adds to the growing evidence that oral pathogens can be primary etiologies of lung abscess, especially in patients with periodontitis and diabetes.

A literature search was conducted in PubMed and Web of Science using the following keywords: “lung abscess”, “pulmonary infection”, “respiratory tract infection”, “*Treponema denticola*”, “*Porphyromonas gingivalis*”, “*Tannerella forsythia*”, and “red complex bacteria”. We included case reports and case series published in English between January 1, 2010 and December 31, 2025, describing lung abscess or pulmonary infection in which at least one red complex bacterium was identified as the causative pathogen. Extracted data included demographics, predisposing factors, clinical presentation, diagnostic method, treatment regimen, and outcomes. Two authors (Zhiyang Xu and Jinliang Liu) independently collected these articles and evaluated their eligibility based on the title and abstract.

Our literature review identified 19 similar cases published since 2010. All cases were diagnosed using mNGS performed on thoracic samples. A history of periodontitis was either explicitly reported or could be inferred from poor oral hygiene in nearly all cases. Among the 19 patients included (Table 1), the average age was 59.3 years (range, 46–77 years), and 74% were male, consistent with a previous study (7). The most common comorbidity among the patients was hypertension (7/19, 36.8%), followed by diabetes mellitus (5/19, 26.3%), also consistent with a previous study (8). The most common clinical presentations comprised cough and sputum production (18/19, 95%), hemoptysis (14/19, 74%), and fever (9/19, 47%). Chest pain and dyspnea were less frequent (5/19 and 3/19, respectively). Laboratory findings often showed a mildly elevated WBC count (mean 10.6 × 10^9^/L) and a marked elevation in CRP (mean 45.8 mg/L). Acavity (15/19, 78.9%) and mass-like lesion (13/19, 68.4%) were usually observed in chest CT. Consolidation and pleural effusion were also noted, and were predominantly located in the right lung, which aligns with findings from other studies (9, 10). Regarding the therapy, the most used regimens after discharge included respiratory fluoroquinolones (9/19), amoxicillin-clavulanate (7/19), and metronidazole (4/19). We defined cured as clinical improvement, such as symptom relief, together with radiological improvement of the lung lesion during follow-up. According to this definition, 18 out of 19 patients (95%) attained clinical and radiological cures. One patient did not meet this definition, attributable to the patient's concurrent severe infections. Clinical information about the 19 patients was listed in Table 2.

**Table 1:** Summary of published case reports of lung abscess involving red complex bacteria (2010–2025).

Study	Year	Pathogens
A Pulmonary Abscess Caused by *Porphyromonas gingivalis* Infection: a Case Report and Literature Review (6)	2025	*Porphyromonas gingivalis*
Clinical characteristics of *Treponema denticola*-associated lung abscess diagnosed by metagenomic next-generation sequencing: a case series analysis (11)	2025	*Porphyromonas, Treponema, Prevotella Streptococcus*
		*Treponema, Tannerella, Parvimonas*
		*Treponema, Tannerella, Porphyromonas*
		*Porphyromonas, Treponema, Tannerella*
		*Treponema*
		*Treponema, Prevotella*
		*Treponema, Porphyromonas, Streptococcus*
*Treponema denticola* as a potential pathogen of pneumonia in individuals with poor oral hygiene: a case report (16)	2025	*Treponema denticola*
Metagenomic Next-Generation Sequencing Reveals *Tannerella forsythia* in Lung Abscesses: a Retrospective Case Series Linking Smoking, Oral Health, and Diagnostic Challenges (8)	2025	*Tannerella forsythia, Porphyromonas gingivalis*
		*Tannerella forsythia, Porphyromonas endodontalis, Treponema socranskii*
		*Tannerella forsythia, Porphyromonas gingivalis, Treponema socranskii*
		*Tannerella forsythia, Porphyromonas endodontalis*
A pulmonary abscess caused by *Porphyromonas endodontalis* infection:A case report and literature review (17)	2024	*Porphyromonas endodontalis, Parvimonas micra*
Lung abscess caused by the anaerobic pathogen *Tannerella forsythia* (18)	2024	*Treponema, Porphyromonas gingivalis, and Tannerella forsythia*
Periodontal Disease as a Possible Cause of a Lung Abscess: a Case Report (19)	2023	*Pseudomonas aeruginosa and Porphyromonas gingivalis*
Lung Abscess Caused by *Tannerella forsythia* Infection: a Case Report (20)	2023	*T. Forsythia, P. gingivalis, Streptococcus milleri and Treponema socranskii*
Subcutaneous abscess due to empyema necessitans caused by *Porphyromonas gingivalis* in a patient with periodontitis (21)	2022	*P. gingivalis*
A community-acquired lung abscess attributable to odontogenic flora (22)	2019	*Porphyromonas_gingivalis, Tannerella_forsythia, Treponema_denticola*

**Table 2:** Clinical characteristics of 19 patients with red complex bacteria lung abscess.

Parameters	Value (*N* = 19)	CT findings	Value (*N* = 19)
Age in Years (mean ± standard deviation)	59.3 ± 8.7	Mass, *n*	13/19
Male/Female, *n*	14/5	Cavity, *n*	15/19
**Smoking**, *n*	9	Consolidation, *n*	6/19
**Drinking**, *n*	6	Pleural effusion, *n*	3/19
**Symptoms**			
Cough/Sputum, *n*	18/19	**Site of involvement**	
Hemoptysis, *n*	14/19	Both, *n*	2/19
Fever, *n*	9/19	Right, *n*	10/19
Chest pain, *n*	5/19	Left, *n*	7/19
Dyspnea, *n*	3/19	**Antibiotics after discharge**	
**Underlying disease**		Quinolones, *n*	9/19
Diabetes mellitus, *n*	5/19	Amoxicillin-clavulanate, *n*	7/19
Hypertension, *n*	7/19	Metronidazole, *n*	4/19
**Laboratory tests**		Doxycycline, *n*	2/19
WBC (^*^10^9^/L, mean ± standard deviation)	10.6 ± 3.5	**Outcome**	
CRP (mg/L, mean ± standard deviation)	45.8 ± 44.2	Cured, *n*	18/19
**Method pathogen detection**		Unhealed, *n*	1/19
BALF NGS, *n*	11/19		
Lung biopsy NGS, *n*	9/19		

Based on the literature and our case, we found that lung abscesses associated with “red complex” bacteria share several distinctive features. Firstly, the symptoms are atypical and prolonged. According to the literature we reviewed, symptom duration ranged from 1 month to approximately 10 months (11). Likewise, our patient had experienced symptoms for 2 months. Secondly, systemic inflammatory responses are relatively mild. The mild inflammatory parameters (normal or slightly elevated inflammatory biomarkers) probably mirror the low virulence and chronic infection patterns typical of the “red complex” bacteria. Third, it is easily misdiagnosed as lung cancer. Radiologically, CT scans of “red complex” associated lung abscesses often show mass-like or solitary lesions with irregular margins and central necrosis, characteristics that are very similar to those of primary lung cancer or metastatic tumors (12). In view of this, our radiologist first considered lung cancer. This overlap can lead to misdiagnosis, delays in appropriate treatment, and unnecessary invasive procedures.

The “red complex bacteria” are strictly anaerobic, fastidious, and part of the oral microbiome (13). In patients with periodontitis, “red complex bacteria” as dominant species co-localize in subgingival plaque (14). The typical biofilm of dental plaque may serve as a reservoir of pathogenic bacteria for respiratory infection. Conventional culture failed to grow red complex bacteria due to their extreme oxygen sensitivity and overgrowth by facultative anaerobes (15). Even targeted anaerobic culture has low yield. mNGS was pivotal in all reviewed cases, highlighting its superiority over conventional culture for detecting these organisms. In the present case, the patient had undergone a prolonged course of antibiotic treatment before referral, which may have contributed to the negative culture results. Routine sputum bacterial culture indicated normal bacterial growth, and sputum tests for aerobic bacteria and anti-acid bacillus cultures yielded negative results. The lung biopsy mNGS provided a clearer signature, pinpointing *T. denticola* and *P. gingivalis* as the causative pathogens. This also prompts us to recognize the necessity of performing NGS testing at the primary infection site when challenges arise in culturing these bacteria on traditional cultures.

The biopsy mNGS results of this patient revealed *Treponema denticola* (1,730 reads, relative abundance 11.1%) and *Porphyromonas gingivalis* (1,374 reads, relative abundance 11.35%) (Figure 2), which were later considered causative pathogen. In addition, Malassezia was detected with a relatively high relative abundance (247 reads, 48.17%). However, the biopsy specimen was obtained through a percutaneous approach, and the simultaneous detection of *Staphylococcus epidermidis*, a common skin commensal, further suggested the possibility of skin-derived background contamination during the sampling procedure. Moreover, there was no clinical, radiological, or pathological evidence supporting fungal lung infection. Therefore, after comprehensive clinical evaluation, Malassezia was interpreted as a background organism. By contrast, the detection of *T. denticola* and *P. gingivalis* in biopsy tissue was consistent with the pathological diagnosis of lung abscess and the patient's severe periodontitis. Taken together, these findings supported red complex bacteria as the most likely causative pathogens in this case.

After the diagnosis was confirmed, the patient received piperacillin-tazobactam followed by amoxicillin-clavulanate and resulted in rapid clinical improvement and radiographic resolution. The excellent response to amoxicillin-clavulanate suggests that oral step-down therapy is effective, provided the isolate is susceptible. The addition of chlorhexidine mouthwash addresses the oral reservoir. In our literature review, most patients achieved cure, but one with severe comorbidities did not, emphasizing the need for prompt diagnosis and comprehensive management. The optimal duration of therapy should be guided by clinical and radiological response.

This study has limitations: the retrospective nature of the literature review, potential publication bias, and lack of standardized reporting. We cannot completely exclude the role of co-pathogens below the detection threshold of mNGS, though the high relative abundance of red complex sequences strongly supports their primary pathogenic role.

Collectively, our findings suggest that red complex bacteria should be considered in patients with lung abscess and periodontitis, particularly when conventional cultures are negative. mNGS should be employed early to guide targeted therapy.

## Conclusion

In summary, the red complex bacteria ought to be considered during the differential diagnosis of culture-negative lung abscess, particularly among patients with periodontal disease. mNGS conducted on lesional tissue represents the preferred diagnostic approach. Elevated clinical awareness and additional prospective investigations are necessary to delineate the optimal management strategies, encompassing the potential function of adjunctive periodontal therapy in preventing recurrence.

## Data Availability

The original contributions presented in the study are included in the article/supplementary material, further inquiries can be directed to the corresponding author.
